# Psychiatric Disorders and Distal 21q Deletion—A Case Report

**DOI:** 10.3390/ijerph17093096

**Published:** 2020-04-29

**Authors:** Wolfgang Briegel, Juliane Hoyer

**Affiliations:** 1 Department of Child and Adolescent Psychiatry, Psychosomatics and Psychotherapy, Leopoldina Hospital, 97422 Schweinfurt, Germany; 2 Department of Child and Adolescent Psychiatry, Psychosomatics and Psychotherapy, University of Würzburg, 97080 Würzburg, Germany; 3 Institute of Human Genetics, University Hospital Erlangen, Friedrich-Alexander-Universität Erlangen-Nürnberg (FAU), 91054 Erlangen, Germany; juliane.hoyer@uk-erlangen.de

**Keywords:** chromosome 21, distal deletion, 21q22.2-q22.3, oppositional defiant disorder, attention deficit/hyperactivity disorder, Parent–Child Interaction Therapy (PCIT), case report

## Abstract

Partial deletion of chromosome 21q is a very rare genetic condition with highly variable phenotypic features including heart defects, high or cleft palate, brain malformations (e.g., cerebral atrophy), developmental delay and intellectual disability. So far, there is very limited knowledge about psychiatric disorders and their effective treatment in this special population. To fill this gap, the authors present the case of an initially five-year-old girl with distal deletion (del21q22.2) and comorbid oppositional defiant disorder (main psychiatric diagnosis) covering a period of time of almost four years comprising initial psychological/psychiatric assessment, subsequent treatment with Parent–Child Interaction Therapy (PCIT), and follow-up assessments. Post-intervention results including a 19-month follow-up indicated good overall efficacy of PCIT and high parental satisfaction with the treatment. This case report makes a substantial contribution to enhancing knowledge on psychiatric comorbidity and its effective treatment in patients with terminal 21q deletion. Moreover, it emphasizes the necessity of multidisciplinarity in diagnosis and treatment due to the variety of anomalies associated with 21q deletion. Regular screenings for psychiatric disorders and (if indicated) thorough psychological and psychiatric assessment seem to be reasonable in most affected children, as children with developmental delays are at increased risk of developing psychiatric disorders. As demonstrated with this case report, PCIT seems to be a good choice to effectively reduce disruptive behaviors in young children with partial deletion of chromosome 21q.

## 1. Introduction

Partial deletion of chromosome 21q (ORPHA574) is a very rare genetic condition (<1/1,000,000) with highly variable phenotypic features, depending on the size and position of the deletion [1,2]. So far, no identical breakpoints have been reported in the literature [3,4]. Three distinct deletion regions have been originally proposed: Region 1 ranging from the centromere to approximately 31.2 Mb (21q11.2-q22.11; about 50 genes), region 2 (31.2–36 Mb, 21q22.11-q22.12; about 80 genes), and region 3 (from 36–37.5 Mb to the telomere, 21q22.12-q22.3; more than 130 genes) [4]. Recently, Errichiello and colleagues have suggested to subset region 1 into two smaller sub-regions: part 1 from the centromere to approximately 21 Mb (21q21.1), and part 2 until approximately 32 Mb, thus including a portion of the 21q22.11 band [2].

Typical clinical features of 21q deletion comprise developmental delay, intellectual disability, growth retardation, microcephaly, brain malformations (e.g., cerebral atrophy), neonatal seizures, heart defects, clinodactyly, high or cleft palate, scoliosis, and dysmorphic facial features such as downward slanting palpebral fissures, broad or depressed nasal bridge, and epicanthal folds [1,2,3,4]. Most cases of deletions in region 1 and 2 have been found to be associated with a severe phenotype, whereas phenotypes of hemizygous deletions in region 3, the most common abnormality, are relatively mild [2,3]. However, a comparison of the phenotypes is difficult, as many cases with partial 21q deletion also show rearrangements including translocations, deletions, or duplications involving other chromosomes [3,4,5].

So far, most studies and case reports on partial deletion of chromosome 21q have primarily focused on genetic and physical aspects. Developmental delays (e.g., speech, language and coordination) and intellectual disabilities are well-known risk factors for the development of psychiatric disorders [6,7] and have been reported to be common problems in patients with this deletion [1,2]. However, only very few cases of partial 21q deletion and specific comorbid psychiatric disorders (based on criteria of the Diagnostic and Statistical Manual (DSM), e.g., [8]) have been described until now [2,9,10,11,12,13], and in most of these cases psychiatric assessment has not been given in detail. Specifically, the following psychiatric disorders have been reported: autism spectrum disorder (ASD) [12,13], schizophrenia [10,11], psychotic episode [2,9], attention-deficit/hyperactivity disorder (ADHD) [12], obsessive–compulsive disorder [2], and major depression [2,13]. Behavioral issues with inattentive, hyperactive, oppositional, and aggressive behaviors have been described for further cases, but without assignment to a specific psychiatric diagnosis [10,14,15]. Almost all reports on specific psychiatric disorders have been on deletions in region 1 [2,9,10,12,14]. Reporting on five own clinical cases and reviewing the literature, Errichiello et al. concluded that deletions in part 1 of region 1 are predominantly associated with intellectual disabilities, whereas they suggested a tighter association between deletions of part 2 and neurobehavioral disorders like obsessive–compulsive disorders, poor social interactions, and vulnerability to psychosis [2]. An association with schizophrenia could not be confirmed by a multi-stage genome-wide association study [16], whereas linkage studies supported evidence for a bipolar affective disorder locus on chromosome 21q22 [17,18,19]. With regard to ASD, studies on a linkage to chromosome 21q have revealed controversial results [20,21].

So far, there have been three reports on the treatment of psychiatric disorders in patients with 21q deletions [9,10,13]. Takhar et al. shortly stated that their 16-year-old patient’s psychotic episode was “treated successfully with an atypical antipsychotic agent” and that “the patient was in full remission after eight weeks of treatment” [9], p. 72. In contrast, the schizophrenic patient whom Murtagh and others described continued to have persistent auditory hallucinations “despite trials of typical and atypical antipsychotic medications” [10], p. 353. Orru and colleagues who presented the case of a 12-year-old male patient with deletion and duplication in the 21q22.3 region reported that combined educational and pharmaceutical treatment (risperidone, 0.25 mg twice a day) followed the DSM-V based diagnosis of ASD [13]. No information on treatment effects have been given by them.

To sum it up, there is very limited knowledge about specific psychiatric disorders in patients with partial deletion of chromosome 21q, especially with regard to the most common deletions affecting region 3. Moreover, apart from one case report [9], information about effective treatment of psychiatric disorders in patients with 21q deletion is completely lacking.

To fill these gaps, the authors present the first report on psychiatric disorders in a patient with a pure distal deletion of chromosome 21q. This case report covers a period of time of almost four years comprising initial psychological/psychiatric assessment, subsequent treatment with Parent–Child Interaction Therapy (PCIT), an evidence-based intervention designed for families with 2 to 6-year-old children with disruptive behavior problems [22], and follow-up assessments.

## 2. Case Presentation

Written informed consent was obtained from both parents for the case report, and this case report was approved by the Ethics Committee of the Bavarian State Medical Association (2020-1086).

In accordance with actual German child and adolescent psychiatry and psychotherapy guidelines [23], assessment comprised a structured and detailed child and family history, psychopathology assessment, physical examination, intelligence testing, administration of disorder-specific questionnaires, and interaction analysis.

### 2.1. Patient Presentation

The patient was a 5 years and 9 months old Caucasian female of average socioeconomic status and a rural upbringing who was referred by the public health department for psychiatric evaluation. Her parents described her as open-minded, but stubborn and never accepting a “no”. They added that she nearly always tried to dominate adults and other children, showing verbally and/or physically aggressive behavior or whining when she did not get what she wanted. Both parents, but especially the mother, the girl’s primary caregiver, reported high levels of parenting stress due to the girl’s challenging behaviors.

### 2.2. Personal History and Family Environment

The patient was born at 36 + 6 weeks’ gestation via C-section due to cardiotocographic abnormalities. She had low birth weight (2090 g, <3rd percentile), height (44.5 cm, 3rd–10th percentile) and head circumference (30 cm, <3rd percentile), and an Apgar score of 7/8/8. After birth she was admitted to a premature infant ward where she was diagnosed with bilateral cleft palate, bilateral cervical cysts, bilateral dacryostenosis, atrial septal defect, right-sided pes calcaneus, and some minor dysmorphic features (clinodactyly, accessory auricle on the left side, bilateral inverted mamilla). Additionally, she suffered from generalized spastic movement disorder and from newborn jaundice, which was successfully treated with phototherapy. Electrocardiogram, electroencephalogram, abdominal and cranial ultrasound, as well as screening for inherited metabolic disorders did not reveal any further abnormalities.

Karyotype and fluorescence in situ hybridization analyses were done at the age of 13 months and revealed a distal 21q deletion (21q22.2-qter deletion). Multiplex ligation-dependent probe amplification analyses showed neither additional microdeletions nor subtelomeric rearrangements. To determine the breakpoints, chromosomal microarray analysis with CytoScan HD Array (Affymetrix^®^, Santa Clara, CA, USA) was conducted which showed a terminal deletion of 4.152 kb in the 21q22.3 region (43,945,335–48,097,372–GRCh37/hg19). The karyotypes of both biological parents were normal.

Because of the generalized spastic movement disorder, the patient had received physiotherapy since the age of two weeks. Speech and occupational therapy had started at the age of two years when speech and motor delays became apparent. Additionally, the girl attended an institution specialized in providing education for children with developmental speech and language delays since the age of four years. Stressful life events had been several operations during childhood (cleft palate, cervical cysts, dacryostenosis, atrial septal defect, pes calcaneus). At clinical presentation, the patient did not take any medication.

The patient was the only child of her non-consanguineous parents who lived together. Both parents had graduated from German “Realschule” (grades 5–10) as an intermediate school after elementary school, which is supposed to prepare mainly practically and theoretically oriented students for trade, technical, and administrative professions. Both parents worked as teachers. While the patient’s father reported no history of mental disorder, her mother reported severe problems with her family of origin (e.g., violence between parents, rejection by her own mother). She described mood swings and feelings of anger, especially towards her daughter, but could not name a specific psychiatric disorder. Moreover, she had already participated in individual behavior therapy without any significant changes.

### 2.3. Pretreatment Assessment

On physical examination, the patient was in good general condition, her height, weight, and head circumference were between the 3rd and 10th percentile, respectively. She showed minor dysmorphic features (see above) and irritation-free scars after surgery.

Unstructured psychiatric interviews with the patient’s parents and her teacher indicated predominantly oppositional defiant behavior problems and attention-deficit/hyperactivity symptoms. During mental examination (based on the Clinical Assessment Scale for Child and Adolescent Psychopathology-D [24]), the patient displayed significant articulation problems combined with rhinolalia aperta. She was cooperative, but to a certain extent inattentive, hyperactive, and impulsive. Semi-structured dyadic parent–child interaction analysis with the Heidelberger Marschak Interaction Method [25] showed very dominant and partly insulting child behaviors towards the mother.

An assessment of cognitive functioning with the German version of the Wechsler Preschool and Primary Scale of Intelligence, 3rd edition [26] revealed a full-scale IQ of 83 (95% confidence interval: 77–91; verbal IQ: 93; performance IQ: 84; processing speed quotient: 82; general language composite: 89). A standardized and validated German instrument to assess different developmental domains (cognitive, emotional, social, language, and fine and gross motor development) in children ages six months to six years (the Entwicklungstest von 6 Monaten bis 6 Jahren [27]), indicated significant developmental delays in expressive language, gross motor skills, and action planning.

The German version of the Eyberg-Child Behavior Inventory (ECBI [28]; for a detailed description see section outcome measures) showed clinically significant disruptive behavior problems (mother ratings: intensity scale: *t*-score: 79; problem scale: *t*-score: 62; father ratings: intensity scale: *t*-score: 74, problem scale: *t*-score: 70). On a well validated German parent and teacher questionnaire on oppositional defiant disorder (ODD) and conduct disorder (CD) symptoms in children (the Fremdbeurteilungsbogen für den Störungsbereich Störungen des Sozialverhaltens [29]), the patient’s teacher reported results conspicuous for ODD (ODD symptoms: percentile: 90–96). A corresponding parent and teacher questionnaire on ADHD symptoms in preschool children, the Fremdbeurteilungsbogen ADHS im Vorschulalter [29], suggested predominantly attention deficits (percentiles: mother ratings: attention deficit: 97–100; hyperactivity–impulsivity: 97–100; total ADHD score: 97–100; father ratings: attention deficit: 78–89; hyperactivity–impulsivity: 78–89; total ADHD score: 78–89; teacher ratings: attention deficit: 97–100; hyperactivity–impulsivity: 12–23; total ADHD score: 61–77).

### 2.4. Diagnoses and Recommendations

Altogether, findings were consistent with clinically significant symptoms of ODD (DSM-V [8]: 313.81; main diagnosis), ADHD–inattentive type (DSM-V: 314.00), and developmental coordination disorder (DSM-V: 315.4). As cleft palate and its long-term consequences is an exclusion criterion, neither language (DSM-V: 315.32) nor articulation disorder (DSM-V: 315.39) was diagnosed despite of significant deficits in speech and language development.

In the absence of specific recommendations regarding the management and treatment of psychiatric disorders in patients with partial deletion of chromosome 21q and in accordance with the guidelines of the German societies of child and adolescent psychiatrists [23], it was recommended that the patient and both of her parents participated in Parent–Child Interaction Therapy (PCIT). PCIT is an evidence-based parent management intervention that offers intensive live coaching to improve parent–child interactions and teach parents effective skills to manage their child’s disruptive behavior problems. Both parents agreed to be involved in this intervention. Stimulant medication was discussed as a second-line intervention for ADHD symptoms, but the parents rejected this option. However, they agreed to continue occupational and speech therapy to reduce developmental coordination and speech deficits.

### 2.5. Brief Description of Standard PCIT

PCIT is a manualized parent management program which aims to help parents develop an authoritative parenting style by combining play and behavior therapy approaches [30]. The treatment consists of two phases: the Child-Directed Interaction (CDI) to help parents learn skills to strengthen their relationship with their child and to increase parental warmth, and the Parent-Directed Interaction (PDI) to help parents learn a clearly structured and consistent approach to discipline. Parents are regularly coached in vivo by the therapist while interacting with their child.

PCIT is assessment-driven regularly using the following two measures: the Eyberg Child Behavior Inventory (ECBI [28]) to monitor the course of disruptive child behavior during therapy, and the Dyadic Parent–Child Interaction Coding System (DPICS [31]) to assess parental management skills and guide coaching. Each PCIT phase starts with a teaching session (parents only) and is followed by coaching sessions with both the child and the parents. In CDI, parents are coached to follow their child’s lead in play situations by using the “PRIDE” skills (P for (labeled) praise, R for reflection, I for imitation, D for behavioral description, and E for enjoyment) and by avoiding behaviors that take away the lead from the child (questions, commands, or criticism). Thus, parents learn to give positive attention to prosocial behaviors, whereas attention-seeking negative behaviors are ignored. Moreover, parents are encouraged to model appropriate behaviors for their children. For a positive therapy progress, generalization from play situations to real-life situations and regular homework are essential. Parents can proceed to PDI after having achieved CDI mastery (i.e., 10 behavioral descriptions; 10 reflections; 10 labeled praises; and no more than three questions, commands, and criticisms in total during a five minute DIPCS-coding) and a significant improvement of their relationship with their child. During PDI, the focus is on coaching parents how to give specific, age-appropriate, direct commands if they really want their child to have something done, and how to proceed with positive reinforcement for compliance or a time-out procedure following noncompliance [32]. PCIT is considered successfully completed when parents reach mastery of the CDI and PDI skills, the child’s ECBI intensity score is below a *t*-score of 55, and parents express confidence in managing their child’s behaviors on their own [32].

### 2.6. Outcome Measures

The German version of the Eyberg Child Behavior Inventory (ECBI [28]) was used as the main child outcome measure. The ECBI is a well-validated 36-item parent-report questionnaire designed to assess disruptive behaviors of children aged two to nine on two scales. Each item rating (on a scale of (1) never to (7) always) reflects the parental perception of behavior frequency (summed to obtain the Intensity Scale score; range from 36–252) and whether this behavior is considered problematic or not (problem scale; yes/no answer; range from 0–36). The problem scale is also considered an indicator of parental distress caused by the child behavior. The ECBI was administered to the parents at pretreatment assessment, at the beginning of each PCIT session, and at 7-month and 19-month follow-up assessments.

To determine whether changes in child disruptive behavior (ECBI intensity score) and parental distress (ECBI problem score) exceeded the margin of measurement error, reliable change indices (RCI [33]) were calculated for graduation, 7- and 19-month follow-up assessments. As there are no German test–retest reliability results so far, Dutch results [34] were used to calculate RCIs. Patients with an RCI ≥ 1.96 are considered to be recovered. To determine whether changes were clinically significant, the Jacobson, Roberts, Berns, and McGlinchey criteria [35] were applied, which imply that the child’s score is in the clinically significant range at time one assessment and in the normal range at time two assessment, and that the change in scores from time one to time two is statistically reliable as defined using the RCI.

To assess the patient’s behavior at school, at 19-month follow-up the German version of Achenbach’s Teacher’s Report Form for Ages 6–18 (TRF/6-18R [36]) was administered. This 113-item questionnaire (ratings: 0 for true, 1 for somewhat or sometimes true, and 2 for very true or often true) comprises eight symptom scales (anxious/depressed; withdrawn/depressed; somatic complaints; social problems; thought problems; attention problems; rule-breaking behavior; aggressive behavior) and three global scales (internalizing problems; externalizing problems; total problems). Internalizing problems combines the three scales anxious/depressed, withdrawn/depressed, and somatic complaints, while externalizing problems comprises of the two scales rule-breaking behavior and aggressive behavior. The total problems score represents the sum of all the problem items. Scores of the German version can be classed as normal, borderline, or clinical.

The German version of the clinical Dyadic Parent–Child Interaction Coding System (DPICS [37]), a behavioral coding system designed to assess parent–child interactions, was administered to examine changes in parent–child interactions from baseline to 19-month follow-up assessment. The clinical DPICS comprises of nine parent categories (only verbalization categories) and three child categories (only response categories). The DPICS is used to code the behaviors of the parent and the child in different standard situations that vary in the degree of parental control or directiveness required. During Child Led Play (CLP), the parent is asked to follow the child’s lead in play, whereas during Parent Led Play (PLP), the parent is asked to lead the play. Each coding situation lasts five minutes.

The Therapy Attitude Inventory (TAI [38]), a well-validated 10-item measure of parental satisfaction with the process and outcome of parent training, was used to measure parents’ intervention satisfaction (range of raw scores: 0–50). Preliminary data suggest that the German version shows good psychometric properties, with a mean total score of 45.8 [39]. The TAI was administered to both parents at the last follow-up session (i.e., 19 months after PCIT graduation).

### 2.7. Course of Treatment

Due to a lack of therapy capacities, the patient and her parents had to wait about 10 months until PCIT could be started (patient’s age at PCIT start: 6 years and 9 months). At baseline, ECBI intensity scores of both parents (see Table 1) still indicated significant disruptive behavior problems, and DPICS coding revealed that the so-called “don’t skills” (parental commands, questions, and criticism) clearly outweighed the “do skills” (parental praises, behavior descriptions, and reflections). For further information on CDI results see Table 2. Surprisingly, the child was quite compliant during the five minutes of Parent Led Play (PLP); she complied to 62.5% of maternal and 73.3% of paternal commands with an opportunity to comply (so-called “alpha-compliance”).

PCIT took place over a period of 14 months. Throughout the PCIT intervention, the patient had speech and occupational therapy but did not receive any other form of intervention, especially no psychopharmacotherapy. All sessions followed the session outline of the German version of the 1999 PCIT manual [40]. No PCIT adaptation was needed. Coaching was conducted through a one-way mirror using a “bug in the ear” device. After the fifth CDI session, the patient started elementary school at a school for children with speech and language problems.

Four months after starting PCIT, the patient’s parents reached criteria to proceed to PDI. Two months later, they separated and agreed that the child should live alternatingly with both parents. Fortunately, couple conflicts never became an obstacle to PCIT treatment, which the family completed successfully after another seven months (for DPICS scores see Table 2). Although the parents’ separation was a life-changing experience for the young patient, her behavior improved continuously until graduation (see Table 1). However, as the patient still triggered anger reactions from her mother, towards the end of PCIT, the patient’s mother additionally attended four individual sessions on anger management strategies.

Until PCIT graduation, the mother participated in 13 CDI and 16 PDI sessions, whereas the father attended 10 CDI and 14 PDI sessions. Clinically significant ECBI changes were found for both mother and father ratings at graduation (see Table 3).

### 2.8. Follow-ups

For both parents, 7- and 19-month follow-ups could be realized. At 7-month follow-up, parents reported that the now 8 years and 6 months old girl had had velopharyngoplastic surgery four months after graduation resulting in an increase of speech problems and a consecutive intensification of speech therapy. Additionally, the girl seemed to have more attention problems at school. Audiometric assessment did not show any hearing problems. Stimulant medication was discussed again, but was again rejected. As ECBI scores were still in the non-clinical range (see Table 1), it was primarily recommended to continue daily special time.

At 19-month follow-up, paternal ECBI scores were still below a *t*-score of 50 indicating no clinically significant disruptive child behaviors (see Table 1). In line with these results, no borderline or clinical scores could be found for any of the scales of the TRF/6-18 which was filled out by the patient’s second grade teacher. In contrast, the mother’s ECBI Intensity Scale score was again above the clinical cut-off (see Table 1), and maternal ECBI ratings now no longer met criteria for a clinically significant change (see Table 3). The patient’s mother reported that she still experienced significant anger or sadness due to her child’s behavior and that her relationship with her own mother remained very problematic. Although the patient’s mother practiced special time only once or twice a week with her daughter and the father had completely stopped special time, both parents still showed significant improvement of CDI skills compared to baseline assessment with the father even fulfilling CDI mastery criteria (see Table 2). In contrast to the patient’s mother, he also demonstrated PDI mastery skills during PLP (100% vs. 60% of correct follow-through). Moreover, the patient complied to 80.0% of maternal and to 100% of paternal commands with an opportunity to comply indicating a significant increase of alpha-compliance compared to baseline assessment (baseline rates: mother: 62.5%; father: 73.3%). In line with these positive findings TAI total scores (mother: 45, father: 44) were similarly high as in other studies on PCIT [38,39].

As the patient’s mother still had significant problems with emotion regulation individual therapy was recommended to her.

## 3. Discussion

Partial deletion of chromosome 21q is a very rare genetic condition with a widely heterogeneous phenotype [1,2]. So far, most studies have focused on genetic and physical aspects, while there is very limited knowledge about specific psychiatric disorders and their effective treatment, especially in subjects with a distal deletion.

We present the case of an initially 5-year-old girl with a de novo deletion of chromosome 21q (del21q22.2-qter) who showed neither additional microdeletions nor subtelomeric rearrangements. In line with findings that hemizygous deletions in region 3 of chromosome 21q are associated with relatively mild phenotypic anomalies [1,2,3], the patient demonstrated the following minor abnormal conditions at birth: low birth weight and head circumference, bilateral cleft palate, bilateral cervical cysts, bilateral dacryostenosis, atrial septal defect, right-sided pes calcaneus, and some minor dysmorphic features (clinodactyly, accessory auricle on the left side, bilateral inverted mamilla). From the age of two years, speech and motor delays became apparent while IQ testing at the age of five years revealed a full-scale IQ in the borderline range between average and below average. Results of psychiatric assessment and well validated psychometric instruments were consistent with clinically significant symptoms of ODD (main diagnosis), ADHD-inattentive type and developmental coordination disorder.

So far, very few cases of partial 21q deletion and comorbid psychiatric disorders have been reported [2,9,10,11,12,13], among them only one case with ADHD [12] and developmental coordination disorder [2], respectively. ODD combined with 21q deletion has not been described until now. Moreover, there has been only one report on psychiatric disorders and a deletion in region 3 so far [13]. In contrast to our case, a report by Orru and colleagues described not a pure distal deletion, but a combination of deletion and duplication in the 21q22.3 region [13], and their patient satisfied the DSM-V criteria for ASD, anxiety, and severe depression, whereas our patient presented with a completely different psychiatric profile. Thus, our report does not support the authors’ hypothesis that the presence of a susceptibility locus for ASD (mapped in a 200 kb region between *PCNT* and *PRMTS*) might be associated with depression and anxiety [13]. However, the mechanisms through which genetic variants affect brain and behavior are poorly understood. Expressivity of genetic variants is variable, especially for neuropsychiatric phenotypes. Phenotypic expression of rare high-penetrance alleles is modulated by other genetic factors, including rare and common variants [41] or epigenetic regulation [42].

Considering that hemizygous 21q deletions are most commonly located in region 3 [1] and that developmental delays, especially language and speech delays, have been reported to be common among patients with partial deletion of chromosome 21q [1,2,4], the rarity of reports on psychiatric disorders in patients with a deletion in region 3 is astonishing and might be best explained by the fact that the main focus has not yet been on psychiatric problems or disorders. In line with this, most reports on 21q deletion and DSM-based psychiatric comorbidity assessment instruments (e.g., intelligence tests or other validated psychometric tools) have not been described in detail. Moreover, with the exception of one case of developmental coordination disorder [2] neither DSM criteria nor validated specific assessment instruments have been applied in the past for the diagnosis of developmental disorders. An alternative explanation for the rarity of reports might be that psychiatric disorders are definitely not present if 21q deletion is diagnosed at a very young age, but that they occur later in life.

Disruptive behavior disorders such as ODD and ADHD (two of our patient’s diagnoses), share a multifactorial pathogenesis [8]. Moreover, to the authors’ best knowledge, linkage studies have not suggested a possible association between 21q deletion and ADHD or ODD so far. Therefore, a causal relationship between 21q deletion and ADHD or ODD in our patient seems to be very questionable. Anyway, disruptive disorders represent one of the most relevant classes of problems affecting children less than eight years of age [8,43]. Without adequate interventions they exhibit considerable stability [44,45]. Moreover, they are associated with profound disability and an increased risk for later life psychopathology and family dysfunction [46]. Therefore, early diagnosis and treatment of disruptive disorders and comorbid problems (like developmental disorders, e.g., language or coordination disorders) is crucial [47]. Most guidelines recommend that psychosocial interventions, especially parent management trainings, constitute first-line treatment for preschool children with disruptive behavior disorders (e.g., [23,48]). The largest effects have been reported for behavioral interventions [49]. As PCIT belongs to the best-evaluated interventions for disruptive behavior disorders [22,50], PCIT was recommended to the patient’s parents. Both parents agreed to be involved in this intervention. In contrast, they repeatedly rejected a trial with stimulant medication.

This is the first report on the effects of a parent management intervention in a patient with 21q deletion. According to the parents’ reports, clinically significant changes regarding disruptive child behaviors could be achieved until graduation and 7-month follow-up, although parents separated during treatment and the patient had to undergo velopharyngoplastic correction after PCIT graduation. At 19-month follow-up, the patient’s father and her elementary school teacher reported no clinically significant disruptive child behaviors, whereas the ratings of the patient’s mother suggested a relapse of ODD. Additionally, DPICS observations revealed positive changes in parent–child interactions from baseline to 19-month follow-up assessment, and parents were very satisfied with PCIT. To sum it up, post-intervention results indicated good overall efficacy of PCIT, similar to studies on disruptive disorders in children from the general population [22] and in children with intellectual disabilities [51] and neurological disorders [52]. Thus, this case report supports recommendations of Unique, the British Rare Chromosome Disorder Support Group, for families with a child with 21q deletion and behavior problems [53], p 12.

Despite clear strengths, including well standardized and carefully chosen clinically relevant measures (comprising parent and teacher reports as well as standardized therapist observations) and long-term post-treatment assessment, this study has some limitations. Methodological limitations inherent in case study designs are the lack of a control group and limited generalizability. In addition, the following specific limitations of the current case study are important to consider. First, neither at pre-treatment nor at follow-up assessment a standardized diagnostic interview for psychiatric disorders in children could be used, although this might have increased the validity of diagnostic findings. The reasons are that there is no validated German interview on psychiatric disorders in preschool children so far, and that an interview for psychiatric disorders in older children at follow-up seemed too time-consuming for the parents. Second, due to age restraints, different teacher rating forms had to be used at pre-treatment and 19-month follow-up assessments. It would have been helpful to add confidence to outcome results and further validate the reported treatment effects, if identical teacher ratings could have been included. Third, towards the end of treatment another intervention component (i.e., four sessions of anger management for the girl’s mother) was implemented concurrently with PCIT. Although this is common in clinical practice, it results in uncertainty about the extent to which this intervention might have contributed to overall improvements at post-intervention. Fourth, no standardized information about the development of parental psychopathology (especially maternal depressive symptoms), the quality of parental relationship, and parenting attitudes have been gathered across pre-treatment and post-intervention assessments. Fifth, no magnetic resonance imaging could be done, which might have uncovered relevant intra-cranial abnormalities.

## 4. Conclusions

Usually, families with a child with partial deletion of chromosome 21q have to face a variety of challenges. For most of the affected families, a crucial aspect is the lack of knowledge on aetiopathogenesis and prognosis of both physical and psychological problems and their effective treatment. As 21q deletion normally results in a complex condition, it seems questionable whether evidence-based standard interventions are equally effective in patients with this deletion as in the general population. This case report makes a substantial contribution to enhancing knowledge on psychiatric comorbidity and its effective treatment in patients with terminal 21q deletion. Moreover, it emphasizes the necessity of multidisciplinarity in diagnosis and treatment due to the variety of anomalies associated with 21q deletion. Regular screenings for psychiatric disorders and (if indicated) thorough psychological and psychiatric assessment seem to be reasonable in most affected children, as children with developmental delays are at increased risk of developing psychiatric disorders [6]. In the absence of specific recommendations, interventions for psychiatric disorders should be chosen in accordance with the guidelines of the psychological and psychiatric scientific societies. As demonstrated with this case report, PCIT seems to be a good choice to effectively reduce disruptive behaviors in young children with partial deletion of chromosome 21q and comorbid ODD and ADHD. Last but not least, similar to other rare genetic conditions without the possibility of a causal therapy, the management and treatment of children with this deletion should comprise early access to developmental therapies such as speech and occupational therapy.

## Figures and Tables

**Table 1:** Eyberg-Child Behavior Inventory (ECBI) scores (mother and father ratings) at baseline, graduation, 7- and 19-month-Follow-up.

ECBI Scales	Baseline: Raw Score (*t*-Score)		Graduation: Raw Score (*t*-Score)		7-Month FU ^1^: Raw Score (*t*-Score)		19-Month FU ^1^: Raw Score (*t*-Score)	
	Mother	Father	Mother	Father	Mother	Father	Mother	Father
Intensity Scale	140 (74)	146 (76)	80 (46)	75 (44)	108 (59)	84 (48)	120 (64)	86 (49)
Problem Scale	19 (70)	19 (70)	0 (40)	3 (44)	5 (48)	6 (49)	10 (56)	3 (44)

^1^ FU: Follow-up.

**Table 2:** Parents’ Child-Directed Interaction (CDI) skills during Child Led Play at baseline, graduation, and 19-month follow-up.

DPICS ^1^	Mother			Father		
	Baseline	Graduation	19-Month FU ^2^	Baseline	Graduation	19-Month FU ^2^
**Do skills**						
Behavioral description	0	14	13	1	17	12
Reflection	3	7 ^3^	4	5	14	14
Labeled praise	2	18	6	1	16	10
Unlabeled praise	2	4	4	2	2	4
**Total**	7	43	27	9	49	40
**Don’t skills**						
Question	17	0	3	23	0	1
Command	3	0	2	7	2	0
Criticism	1	0	0	1	1	0
**Total**	21	0	5	31	3	1

^1^ DPICS: Dyadic Parent-Child Interaction Coding System. ^2^ FU: Follow-up. ^3^ More than 80% of child statements that could be reflected were reflected.

**Table 3:** Reliable Change Indices and Clinically Significant Changes at graduation, 7- and 19-month follow-up.

Type of Comparison	ECBI-Scale	Mother Rating		Father Rating	
		RCI ^1^	CSC ^2^	RCI ^1^	CSC ^2^
Pretreatment vs. Graduation	Intensity	−4.64	YES	−5.47	YES
	Problem	−3.20	YES	−2.77	YES
Pretreatment vs. 7-month FU ^3^	Intensity	−2.47	YES	−4.78	YES
	Problem	−2.36	YES	−2.25	YES
Pretreatment vs. 19-month FU ^3^	Intensity	−1.55	NO	−4.63	YES
	Problem	−1.52	NO	−2.77	YES

^1^ RCI = Reliable Change Index; RCIs > 1.96 are statistically significant. ^2^ CSC = Clinically Significant Change. ^3^ FU = Follow-up.

## References

[1] Roberson E.D., Wohler E.S., Hoover-Fong J.E., Lisi E., Stevens E.L., Thomas G.H., Leonard J., Hamosh A., Pevsner J. (2011). Genomic analysis of partial 21q monosomies with variable phenotypes. Eur. J. Hum. Genet..

[2] Errichiello E., Novara F., Cremante A., Verri A., Galli J., Fazzi E., Bellotti D., Losa L., Cisternino M., Zuffardi O. (2016). Dissection of partial 21q monosomy in different phenotypes: Clinical and molecular characterization of five cases and review of the literature. Mol. Cytogenet..

[3] Meng S., Benke P.J., Bademci G., Cengiz F.B., Ouyang X., Peng J., Casas C.E., Tekin M., Fan Y.-S. (2018). Monosomy chromosome 21 compensated by 21q22.11q22.3 duplication in a case with small size and minor anomalies. Mol. Cytogenet..

[4] Lyle R., Béna F., Gagos S., Gehrig C., Lopez G., Schinzel A., Lespinasse J., Bottani A., Dahoun S., Taine L. (2009). Genotype-phenotype correlations in Down syndrome identified by array CGH in 30 cases of partial trisomy and partial monosomy chromosome 21. Eur. J. Hum. Genet..

[5] Jespersgaard C., Damgaard I.N., Cornelius N., Bache I., Knabe N., Miranda M.J., Tümer Z. (2016). Proximal 21q deletion as a result of a *de novo* unbalanced t(12;21) translocation in a patient with dysmorphic features, hepatomegaly, thick myocardium and delayed psychomotor development. Mol. Cytogenet..

[6] Strømme P., Diseth T. (2000). Prevalence of psychiatric diagnoses in children with mental retardation: Data from a population-based study. Dev. Med. Child Neurol..

[7] Emerson E. (2003). Prevalence of psychiatric disorders in children and adolescents with and without intellectual disability. J. Intellect. Disabil. Res..

[8] American Psychiatric Association (2013). Diagnostic and Statistical Manual of Mental Disorders.

[9] Takhar J., Mall A.K., Siu V., MacPherson C., Fan Y.S., Townsend L. (2002). An interstitial deletion of the long arm of chromosome 21 in a case of a first episode of psychosis. Acta Psychiatr. Scan..

[10] Demirhan O., Tastemir D. (2003). Chromosome aberrations in a schizophrenia population. Schizophr. Res..

[11] Murtagh A., McTigue O., Ramsay L., Hegarty A.M., Green A.J., Stallings R.L., Corvin A. (2005). Interstitial deletion of chromosome 21q and schizophrenia susceptibility. Schizophr. Res..

[12] Haldeman-Englert C.R., Chapman K.A., Kruger H., Geiger E.A., McDonald-McGinn D.M., Rappaport E., Zackai E.H., Spinner N.B., Shaikh T.H. (2010). A de novo 8.8-Mb deletion of 21q21.1-q21.3 in an autistic male with a complex rearrangement involving chromosomes 6, 10, and 21. Am. J. Med. Genet. A.

[13] Orru O., Papoulidis I., Siomou E., Papadimitriou D.T., Sotiriou S., Nikolaidis P., Eleftheriades M., Papanikolaou E., Thomaidis L., Manolakos E. (2019). Autism spectrum disorder, anxiety and severe depression in a male patient with deletion and duplication in the 22q22.3 region: A case report. Biomed. Rep..

[14] Petit F., Plessis G., Decamp M., Cuisset J.M., Blyth M., Pendlebury M., Andrieux J. (2015). 21q21 deletion involving NCAM2: Report of 3 cases with neurodevelopmental disorders. Eur. J. Med. Genet..

[15] Oegema R., de Klein A., Verkerk A.J., Schot R., Dumee B., Douben H., Eussen B., Dubbel L., Poddighe P.J., van der Laar I. (2010). Distinctive phenotypic abnormalities associated with submicroscopic 21q22 deletion including *DYRK1A*. Mol. Syndromol..

[16] Schizophrenia Working Group of the Psychiatric Genomics Consortium (2014). Biological insights from 108 schizophrenia-associated genetic loci. Nature.

[17] Liu J., Juo S.H., Terwilliger J.D., Grunn A., Tong X., Brito M., Loth J.E., Kanyas K., Lerer B., Endicott J. (2001). A follow-up linkage study supports evidence for a bipolar disorder locus on chromosome 21q22. Am. J. Med. Genet..

[18] Kaneva R.P., Chorbov V.M., Milanova V.K., Kosotv C.S., Nickolov K.I., Chakarova C.F., Stoyanova V.S., Nikolova-Hill A.N., Krastev S.K., Onchev G.N. (2004). Linkage analysis in bipolar pedigrees adds support for a susceptibility locus on 21q22. Psychiatr. Genet..

[19] Roche S., Cassidy F., Zhao C., Badger J., Claffey E., Mooney L., Delaney C., Dobrin S., McKeon P. (2007). Candidate gene analysis of 21q22: Support for S100B as a susceptibility gene for bipolar affective disorder with psychosis. Am. J. Med. Genet. B Neuropsychiatr. Genet..

[20] Molloy C.A., Keddache M., Martin L.J. (2005). Evidence for linkage on 21q and 7q in a subset of autism characterized by developmental regression. Mol. Psychiatry.

[21] Parr J.R., Lamb J.A., Bailey A.J., Monaco A.P. (2006). Response to paper by Molloy et al.: Linkage on 21q and 7q in autism subset with regression. Mol. Psychiatry.

[22] Eyberg S.M., Nelson M.M., Boggs S.R. (2008). Evidence-based psychosocial treatments for children and adolescents with disruptive behavior. J. Clin. Child Adolesc. Psychol..

[23] Deutsche Gesellschaft für Kinder- und Jugendpsychiatrie, Psychosomatik und Psychotherapie (Hrsg.) für die Leitliniengruppe: Leitlinien zu Psychischen Störungen im Säuglings-, Kleinkind- und Vorschulalter (S2k-Leitlinie 028/041). https://www.awmf.org/uploads/tx_szleitlinien/028-041l_S2k_Psychische_Stoerungen_Saeugling_Kleinkind_Vorschulalter_2017-10.pdf.

[24] Döpfner M., Berner W., Flechtner H., Lehmkuhl G., Steinhausen H.C. (1999). Psychopathologisches Befund-System für Kinder und Jugendliche (CASCAP-D).

[25] Ritterfeld U., Franke U. (1994). Die Heidelberger Marschak-Interaktionsmethode (H-MIM).

[26] Petermann F., Ricken G., Fritz A., Schuck K.D., Preuß U. (2014). Wechsler Preschool and Primary Scale.

[27] Petermann F., Stein I.A., Macha T. (2008). Entwicklungstest von 6 Monaten bis 6 Jahre (ET 6-6).

[28] Eyberg S.M., Pincus D. (1999). Eyberg Child Behavior Inventory and Sutter Eyberg Student Behavior Inventory—Revised: Professional manual.

[29] Döpfner M., Görtz-Dorten A., Lehmkuhl G. (2008). Diagnostik-System für Psychische Störungen nach ICD-10 und DSM-IV für Kinder und Jugendliche—II (DISYPS-II).

[30] Eyberg S.M. (1988). Parent-child interaction therapy: Integration of traditional and behavioral concerns. Child Family Behav. Ther..

[31] Eyberg S.M., Nelson M.M., Ginn N.C., Bhuiyan N., Boggs S.R. (2013). Dyadic Parent-Child Interaction Coding System (DPICS). Comprehensive Manual for Research and Training.

[32] Eyberg S., Funderburk B. (2011). Parent-Child Interaction Therapy Protocol.

[33] Jacobson N.S., Truax P. (1991). Clinical significance: A statistical approach to defining meaningful change in psychotherapy research. J. Consult. Clin. Psychol..

[34] Abrahamse M.E., Junger M., Leitjen P.H.O., Lindeboom R., Boer F., Lindauer R.J.L. (2015). Psychometric properties of the Dutch Eyberg Child Behavior Inventory (ECBI) in a community sample and a multi-ethnic clinical sample. J. Psychopathol. Behav. Assess.

[35] Jacobson N.S., Roberts L.J., Berns S.B., McGlinchey J.B. (1999). Methods for defining and determining the clinical significance of treatment effects: Description, application, and alternatives. J. Consult. Clin. Psychol..

[36] Döpfner M., Plück J., Kinnen C. (2014). (für die Arbeitsgruppe Deutsche Child Behavior Checklist). CBCL/6-18R, TRF/6-18R, YSR/6-18R. Deutsche Schulalter-Formen der Child Behavior Checklist von Thomas M. Achenbach.

[37] Eyberg S.M., Members of the Child Study Lab (2010). Abbreviated Manual of the Dyadic Parent-Child Interaction Coding System (DPICS). Version 3.09.

[38] Brestan E.V., Jacobs J.R., Rayfield A.D., Eyberg S.M. (1999). A consumer satisfaction measure for parent-child treatment and its relation to measures of child behavior change. Behav Therapy.

[39] Briegel W., Walter T., Schimek M., Knapp D., Bussing R. (2015). Parent-Child Interaction Therapy im In-room-Coaching. Kindh. Entwickl..

[40] Eyberg S. (1999). Members of the Child Study Laboratory. PCIT: Parent-Child Interaction Therapy. Integrity Checklists and Session Materials.

[41] Purcell S.M., Wray N.R., Stone J.L., Visscher P.M., O’Donovan M.C., Sullivan P.F., Sklar P. (2009). Common polygenic variation contributes to risk of schizophrenia and bipolar disorder. Nature.

[42] Hirasawa R., Feil R. (2010). Genomic imprinting and human disease. Essays Biochem..

[43] Egger H.L., Angold A. (2006). Common emotional and behavioral disorders in preschool children: Presentation, nosology, and epidemiology. J. Child Psychol. Psychiatry.

[44] Esser G., Ihle W., Schmidt M.H., Blanz B. (2000). Der Verlauf psychischer Störungen vom Kindes- zum Erwachsenenalter. Z. Klin. Psych Psychoth.

[45] Lavigne J.V., Ciccetti C., Gibbons R.D., Binns H.J., Larsen L., DeVito C. (2001). Oppositional defiant disorder with onset in preschool years: Longitudinal stability and pathways to other disorders. J. Am. Acad Child Adolesc. Psychiatry.

[46] Burke J.D., Waldman I., Lahey B.B. (2010). Predictive validity of childhood oppositional defiant disorder and conduct disorder: Implications for the DSM-V. J. Abnorm. Psychol..

[47] Wakschlag L.S., Briggs-Gowan M.J., Carter A.S., Hill C., Danis B., Keenan K., McCarthy K.J., Leventhal B.L. (2007). A developmental framework for distinguishing disruptive behavior from normative misbehavior in pre-school children. J. Child Psychol. Psychiatry.

[48] Gleason M.M., Egger H.L., Emslie G.J., Greenhill L.L., Kowatch R.A., Liebermann A.F., Luby J.L., Owens J., Scahill L.D., Scheeringa M.S. (2007). Psychopharmacological treatment for very young children: Contexts and guidelines. J. Am. Acad Child Adolesc. Psychiatry.

[49] Comer J.S., Chow C., Chan P.T., Cooper-Vince C., Wilson L.A.S. (2013). Psychosocial treatment efficacy for disruptive behavior problems in very young children: A meta-analytic examination. J. Am. Acad Child Adolesc. Psychiatry.

[50] Herr L., Mingebach T., Becker K., Christiansen H., Kamp-Becker I. (2015). Wirksamkeit elternzentrierter Interventionen bei Kindern im Alter von zwei bis zwölf Jahren. Kindh. Entwickl..

[51] Bagner D.M., Eyberg S.M. (2007). Parent-child interaction therapy for disruptive behavior in children with mental retardation: A randomized controlled trial. J. Clin. Child Adolesc. Psychol.

[52] Shafi R.M.A., Vande Voort J.L., Croarkin P.E., Romanowicz M. (2018). Parent-child interaction therapy in a case of global developmental delay and leukoencephalopathy. Front. Psychiatry.

[53] (2005). Unique (Understanding chromosome disorders): *21q deletions*; Rare Chromosome Disorder Support Group: Oxted, UK. https://www.rarechromo.org/media/information/Chromosome%2021/21q%20deletions%20FTNW.pdf.

